# Sonographic detection of massive colonic pseudopolyposis in inflammatory bowel diseases

**DOI:** 10.1007/s40477-023-00853-w

**Published:** 2024-01-29

**Authors:** Sofia Silva Mendes, Federica Lepore, Mary Hussey, Giovanni Cataletti, Annalisa De Silvestri, Giovanni Maconi

**Affiliations:** 1 Gastroenterology Department, Braga Hospital, Braga, Portugal; 2 School of Medicine, Life and Health Sciences Research Institute (ICVS), University of Minho, Braga, Portugal; 3 Gastroenterology Unit, Luigi Sacco University Hospital, University of Milan, Milan, Italy; 4 Department of Biomedical and Clinical Sciences Luigi Sacco, University of Milan, Milan, Italy; 5 Department of Gastroenterology, University Hospital Galway, Galway, Ireland; 6 Clinical Epidemiology and Biometeric Unit, Fondazione IRCCS Policlinico San Matteo, Pavia, Italy

**Keywords:** Inflammatory bowel diseases, Intestinal ultrasound, Pseudopolyps, Post-inflammatory polyps

## Abstract

**Purpose:**

Colonic pseudopolyps are a frequent finding in inflammatory bowel disease (IBD). Yet there are no published data describing the characteristics of pseudopolyposis in intestinal ultrasound (IUS). This study aimed at identifying the key features of pseudopolyposis in IUS.

**Methods:**

This case–control study included 12 patients with ulcerative colitis or Crohn’s colitis with extensive left colon pseudopolyposis and 18 matched IBD patients without pseudopolyps at colonoscopy. Luminal (diameters, thickening, stratification, margins, and vascularity) and intraluminal (vascular signals at color Doppler), and extraluminal (mesenteric fat) parameters of the left colon were compared. Anonymized still images and videos of these patients were blindly reviewed to estimate the accuracy in detecting this condition.

**Results:**

Among the IUS parameters assessed, the anteroposterior diameter ≥ 12 mm and the presence of luminal vascular signals were significantly correlated with pseudopolyposis. The detection of both these findings were able to detect extensive pseudopolyposis a sensitivity of 75% (CI 95%: 42.8–94.5%) and a specificity of 100% (CI 95%: 81.5–100%).

**Conclusion:**

This is the first study describing the IUS features of pseudopolyposis in IBD. The potential use of IUS to assess pseudopolyposis might have an impact on IUS monitoring and surveillance of IBD patients with condition.

**Supplementary Information:**

The online version contains supplementary material available at 10.1007/s40477-023-00853-w.

## Introduction

Colonic post-inflammatory polyps (or pseudopolyps) are a common finding in inflammatory bowel disease (IBD) both in ulcerative colitis (UC) and in colonic Crohn’s disease (CD), with and estimated incidence of 10–20% [1]. Post-inflammatory polyps are defined endoscopically as cicatricial, digitiform protrusions of the mucosa that result from re-epithelization and excessive regeneration of ulcerated colonic mucosa [1]. They are often multiple and are more frequently found in the left colon [3].

Post-inflammatory polyps are a marker of previous episodes of severe inflammatory activity and several studies have shown an association with an increased risk of colorectal cancer, resulting in the need for shortened surveillance intervals [3, 4]. However, a recent study reported an association between post-inflammatory polyps and increased risk of disease severity, more extensive colonic disease as well as higher rates of colectomy but not with an increased risk of colorectal cancer [5].

The management of IBD patients requires clinical, biochemical, endoscopic, and cross-sectional imaging assessment. Intestinal ultrasound (IUS) is a non-invasive, radiation-free, and inexpensive tool, with excellent patient tolerability. It is now being widely used in the field of IBD and reliably enables disease activity monitoring in both UC and CD patients [6, 7].

To date, there are no data about the IUS features of pseudopolyposis in IBD. Considering the impact of the detection of pseudopolyps amongst IBD subjects has on potential disease risk stratification and potential future surveillance planning as well as increasing dependence on IUS for disease monitoring, our study was aimed at defining the IUS features of pseudopolyposis in IBD in order to improve detection.

## Methods

### Study design

A prospective case–control study was conducted in a tertiary IBD referral center. Adult IBD patients with an established diagnosis of UC or colonic CD affecting at least the left colon who had been referred for routine IUS for disease monitoring were included.

Patients incapable of giving their informed consent were excluded. Due to the known limitations of IUS in detecting sparse small polyps of the colon, we assessed the possibility of defining the presence of extensive pseudopolyposis, a condition without a clear endoscopic definition so far, but commonly considered as the presence of multiple, innumerous, pseudopolyps of variable size, within the colon.

*Cases*: a consecutive series of patients with UC or Crohn’s colitis with extensive pseudopolyposis of the sigmoid and/or descending colon documented at colonoscopy performed in the previous 6 months.

*Controls*: UC or Crohn’s colitis patients without pseudopolyps at previous colonoscopy. These patients had comparable clinical, endoscopic activity and demographic characteristics as the pseudopolyposis group.

### Data collection

For each patient the severity of disease activity was assessed by the partial Mayo score for UC and by Harvey Bradshaw Index for CD [8]. Additionally, the following information were also obtained: age, sex, location of the disease, duration of the disease and current therapy.

The IUS exams were performed in a fasting state, without any colonic preparation, using a standard sonographic machine (Esaote MyLab 70 Gold) with convex low resolution (3–5 MHz) and linear high resolution (5–11 MHz) probes. Conventional intestinal parameters, such as maximum bowel wall thickening, bowel wall echopattern (stratified, partially disrupted stratification and extensive hypoechoic) and intestinal wall vascularization at color Doppler (present or absent) and mesenteric fat hypertrophy (present or absent), were collected as previously reported [6]. Based on IUS expertise and experience at our center, and the few radiology reports in the literature [9], additional parameters potentially correlated with pseudopolyposis were also recorded: colonic anteroposterior diameter; total bowel area; any irregularity of internal margins of the colonic wall; color Doppler signals within the lumen with confirmation at pulsed-Doppler assessment. The latter was assessed with gain and frequency optimization, with velocity scale for color Doppler set at 5 cm/s. IUS features were assessed in cases and controls in the same colonic segments (proximal sigmoid colon/distal descending colon) between the left flank and left iliac fossa avoiding segments full of fecal material with shadowing effect and with visualization of posterior margin hampered by colonic gas. All IUS exams were performed within 6 months (median: 1 month, range: 0–6 months) from the colonoscopy and therapy was kept unchanged between two examinations. Images and cineloops of these segments (at least 3 transverse sections of the bowel, with and without color Doppler evaluation) were recorded, anonymized and read after 1 month. All the data were then entered in an electronic data system.

### Statistical analysis

Statistical analysis was performed using IBM® SPSS® Statistics, version 27, and Microsoft Office Excel. For descriptive statistics, all normally distributed variables were described using mean and standard deviation; for all other variables location measures were used, namely median and interquartile range (IQR). For qualitative variables, absolute and relative frequencies were used for descriptive analysis.

The distribution of variables between cases and controls has been estimated by using Fisher test and Student t test. A *p* value < 0.05 was considered statistically significant.

## Results

Twelve patients with massive pseudopolyposis and 18 matched IBD patients without pseudopolyps were recruited. Their clinical features are summarized in Table 1. Most patients and controls were in clinical and endoscopic remission.

**Table 1:** Clinical demographic parameters of IBD patients with pseudopolyposis (cases) and without pseudopolyps (controls)

Clinical parameters	Cases (*n* = 12)	Controls (*n* = 18)	*P* value
Males, *n* (%)	7 (58%)	9 (50%)	0.72
Ulcerative colitis	6 (50%)	9 (50%)	1.0
Left sided colitis	1 (8%)	3 (17%)	0.63
Extensive colitis	5 (42%)	6 (33%)	0.71
Crohn’s disease	6 (50%)	9 (50%)	1.0
Ileocolic	3 (25%)	4 (22%)	1.0
Colic	3 (25%)	5 (28%)	1.0
Current therapy*			
No therapy	1 (8%)	2 (11%)	1.0
Mesalazine	7 (58%)	6 (33%)	0.26
Steroids	1 (8%)	2 (11%)	1.0
Immunosuppressants	2 (16%)	4 (22%)	1.0
Anti TNF-alpha	0	3 (17%)	0.26
Vedolizumab	1 (8%)	3 (17%)	0.63
Ustekinumab	2 (16%)	2 (11%)	1.0
Clinical Active disease #	5 (42%)	6 (33%)	0.71
Endoscopically active disease§	7 (58%)	9 (50%)	0.72
Age (year), mean (IQR)	52 (42–58)	60 (37–74)	0.50
Disease duration (year)	16 (6–22)	9 (4–21)	0.99
IUS parameters			
Bowel wall thickening (mm)	3.5 (2.6–4.2)	2.7 (2.2–3.5)	0.17
Preserved stratification	8 (67%)	17 (94%)	0.046
Irregular internal margins	4 (33%	0	0.009
Mesenteric fat hypertrophy	5 (42%)	4 (22%)	0.25
Bowel wall vascularity (LS > 0)	9 (75%)	7 (39%)	0.07
Luminal color Doppler signals	9 (75%)	0	< 0.001
Bowel AP diameter (mm)	14.5 (12–16.5)	8.5 (7–11)	< 0.001
Bowel LL diameter (mm)	25 (23.5–25.7)	23 (20–25)	0.21
Bowel transversal area (cm^2^)	259 (235–341)	156 (104–216)	< 0.001
AP/LL diameter	0.61 (0.5–0.69)	0.40 (0.32–0.52)	< 0.001

*IQR* interquartile range, *TNF* tumor necrosis factor, *IUS* intestinal ultrasound, *AP* anteroposterior, *LL* laterolateral, *LS* Limberg score

*Some patients received more than 1 treatment; #UC: partial Mayo score > 1; CD: Harvey Bradshaw index > 4

^§^UC: partial Mayo score > 1; CD: SES-CD > 3

**Fig. 1:**
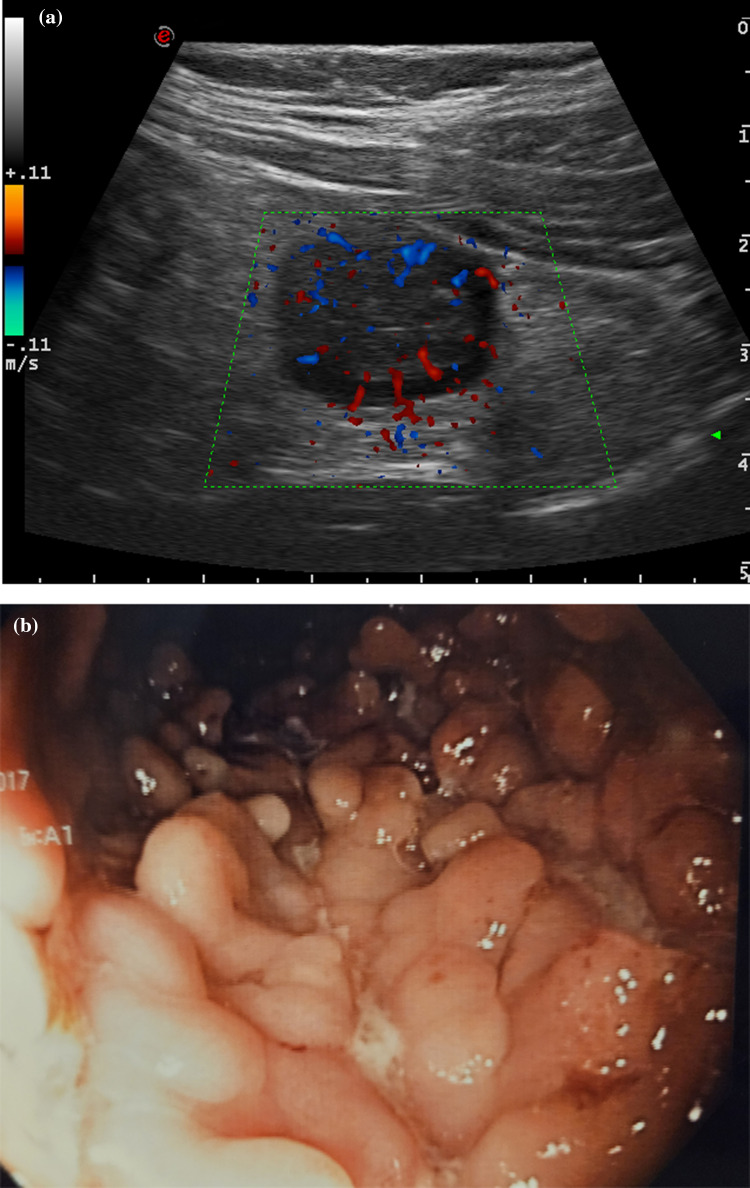
Cross sectional intestinal ultrasound still image (**a**) of the sigmoid in a patients with colonic Crohn’s disease in clinical remission showing massive pseudopolyposis as an increased anteroposterior diameter with color Doppler signals in the wall spreading within the lumen. Endoscopic features of the correspondent sigmoid colon, showing massive pseudopolyposis (**b**)

**Fig. 2:**
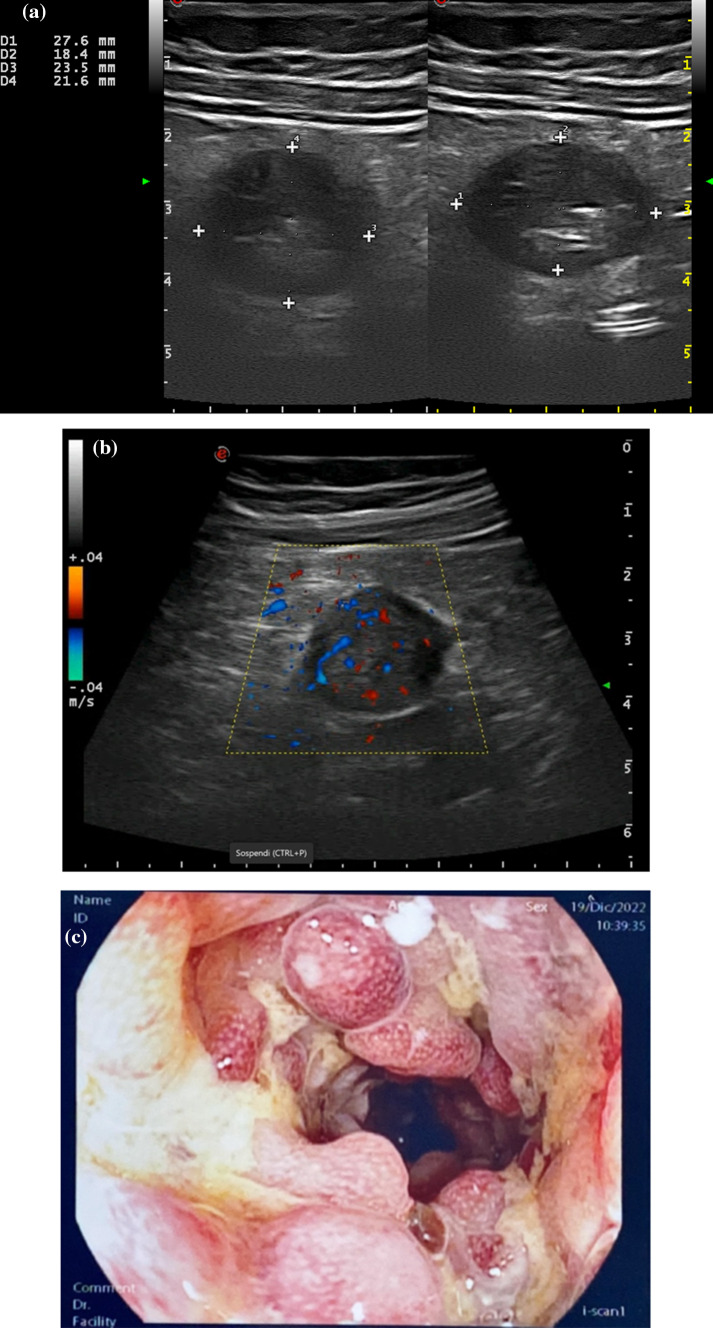
Cross sectional intestinal ultrasound image of the descending colon in a patients with colonic Crohn’s disease in clinical remission showing massive pseudopolyposis as an increased anteroposterior diameter, in two different close segments (**a**) with color Doppler signals spreading within the lumen (**b**, Video 1). Endoscopic features of the correspondent colonic segment showing massive pseudopolyposis (**b**)

The colonic segments with and without massive pseudopolyposis were not significantly different in relation to bowel wall thickening and wall vascularity (Table 1). However, segments with pseudopolyposis showed a significantly larger anteroposterior diameter (14.5 vs 8.5 mm, *p* < 0.001), a greater transversal area (259 vs 156 mm^2^, *p* < 0.001), with a more circular configuration (anteroposterior (AP)/laterolateral (LL) diameter: 0.61 vs 0.40, *p* < 0.001), and vascular signals within the lumen (75% vs 0%) compared with segments of patients without pseudopolyposis (Figs. 1, 2).

The most feasible and assessable variables with the greatest accuracy for assessing massive pseudopolyposis were colonic anteroposterior diameter ≥ 12 mm (lower interquartile value) and the presence of luminal vascular signals. Using the combination of both of these parameters enabled detection of massive pseudopolyposis with a sensitivity of 75% (CI 95%: 42.8–94.5%) and a specificity of 100% (CI 95%: 81.5–100%).

## Discussion

Intestinal ultrasound is a non-invasive tool with increasing evidence of usefulness in the monitoring of CD and UC patients. It allows the assessment of disease activity, extent, and disease-related complications with a potentially significant impact on the management of IBD patients [10].

Colonic pseudopolyps are a frequent finding and a marker of previous inflammatory activity in IBD patients [1]. Importantly, pseudopolyps are considered an intermediate risk factor for colorectal cancer justifying a shortening of the surveillance interval. Moreover, they can make the endoscopic assessment of inflammatory activity and colorectal cancer surveillance in IBD patients difficult due to the irregularity of the mucosa and luminal crowding.

This is the first study describing the IUS features of pseudopolyposis in IBD. We found that increased bowel AP diameter and presence of luminal vascular signals were distinguishing features of pseudopolyposis. Increased colonic AP diameter may be explained by the fact that pseudopolyps usually distend the lumen and make it more circular and likely less compressible, even in patients in endoscopic remission. The size, shape and echogenicity of the lumen may vary according to the amount and consistency of the fecal content and amount of gas in the lumen. As this could be a limitation in the assessment of luminal parameters, we considered only patients where lumen and posterior wall were well visualized, avoiding segments full of solid fecal content and gas.

Color Doppler signals within the lumen of pseudopolyposis patients were also noted. This is likely because pseudopolyps are characterized by a high number of vessels and highly vascularized with slow flow detectable by color Doppler [11]. The use of contrast enhanced US and new technical advances such as the superb microvascular imaging techniques, may better assess pseudopolyposis overcoming some of the limitations of our study. In fact, the sole use of color Doppler fails to detect smaller and sparse pseudopolyps, and can detect artifacts such as the “twinkling artifact” which may result in false positive vascular findings if not confirmed by pulsed-Doppler assessment. This artifact appears as a rapid alternation of colors immediately behind a stationary solid echogenic object (usually a biliary or kidney stone), creating a false image of color flow, which may be visible when colonic lumen is filled by semisolid feces.

Another limitation of our study is that it refers to massive pseudopolyposis of the left colon only. We focused our attention on sigmoid and descending colon because are usually better visualized and more comparable in terms of size amongst IBD patients.

Overall, we observed that the combination of simple anteroposterior diameter of the colon and vascular signals within the lumen suggest a massive pseudopolyposis with high sensitivity and specificity. Given the nature of the study (case–control study) it was not possible to give further information about the accuracy of these IUS signs. Further studies with larger sample and a different study design, should be performed to assess accuracy of IUS in assessing this condition. However, despite these limitations, we feel this study being the first sonographic description of pseudopolyposis in IBD, may be a useful adjunct in the field of IUS in IBD, with potential applications in clinical practice and hints for research. On this regard, although not specifically addressed in our study, the measurement of bowel wall thickening in patients with pseudopolyposis may be tricky due to irregularity and lack of clarity of the mucosal/bowel content interface line. As bowel wall thickening and vascularization are now relevant parameters to assess IBD activity [6], the presence of massive pseudopolyposis could hamper this evaluation and should be therefore reported when suspected. In addition, whether the IUS detection of massive psuedopolyposis could be of add value in clinical practice as prognostic factor or in monitoring IBD patients, remains to be properly assessed in ad hoc studies.

### Supplementary Information

Below is the link to the electronic supplementary material.

Supplementary file1 (MP4 10899 KB)

Supplementary file2 Video 1s. Cross sectional intestinal ultrasound videoclip of the sigmoid colon in a patients with ulcerative colitis with massive pseudopolyposis, characterized by an increased anteroposterior diameter and color Doppler signals spreading within the lumen. (MP4 9590 KB)

Supplementary file3 (DOCX 34 KB)

## Data Availability

Full data are available and may be provided by requests.
